# Outcomes of Percutaneous Coronary Intervention in Patients with Inflammatory Bowel Disease

**DOI:** 10.3390/jcm15062431

**Published:** 2026-03-22

**Authors:** Umesh Bhagat, Akshat Banga, Ankit Agrawal, Prabhat Kumar, Aro Daniela Arockiam, Akiva Rosenzveig, Danial Nasif, Heba Wassif, Jean-Paul Achkar

**Affiliations:** 1 Department of Gastroenterology, Hepatology and Nutrition, Digestive Disease Institute, Cleveland Clinic, Cleveland, OH 44195, USA; bhagatu@ccf.org; 2 Department of Medicine, Mount Auburn Hospital, Harvard Medical School, Cambridge, MA 02115, USA; 3 Department of Cardiovascular Medicine, Heart, Vascular and Thoracic Institute, Cleveland Clinic, Cleveland, OH 44195, USA; aagrawal@uams.edu (A.A.);; 4 Department of Gastroenterology, Virginia Commonwealth University, Richmond, VA 23298, USA

**Keywords:** inflammatory bowel disease, Crohn’s disease, ulcerative colitis, percutaneous coronary intervention

## Abstract

**Background:** Inflammatory bowel disease (IBD), comprising Crohn’s disease (CD) and ulcerative colitis (UC), has been associated with elevated cardiovascular risks. However, the impact of IBD on outcomes following percutaneous coronary intervention (PCI) remains underexplored. We aimed to evaluate the clinical and procedural outcomes of PCI in patients with concurrent IBD. **Methods:** This study utilized the National Readmission Database from 2016 to 2020 to evaluate outcomes such as all-cause mortality and post-PCI complications, including various cardiovascular and gastrointestinal (GI) complications in IBD patients undergoing PCI. Patients with concurrent IBD and PCI were compared to non-IBD controls via multivariable logistic regression. **Results:** On propensity-score-matching analysis, IBD patients undergoing PCI had a higher prevalence of GI complications, including acute liver failure (Odds ratio (OR) 1.48, 95% confidence interval (CI) 1.13–1.93, *p* = 0.004), mesenteric ischemia (OR 5.34, 95% CI 1.56–18.40, *p* = 0.007), and need for blood transfusion (OR 1.74, 95% CI 1.46–2.08, *p* < 0.001). There was also a higher rate of cardiac complications (OR 1.31, 95% CI 1.05–1.64, *p* = 0.017). No significant difference in all-cause mortality (OR 0.86, 95% CI 0.72–1.04, *p* = 0.113) was observed. **Conclusions:** IBD patients undergoing PCI face increased GI and cardiovascular complications without a significant mortality difference. These findings highlight the complex interplay between systemic inflammation, vascular integrity, and procedural outcomes in IBD patients.

## 1. Introduction

Inflammatory bowel disease (IBD), comprising ulcerative colitis (UC) and Crohn’s disease (CD), is a chronic systemic inflammatory condition that affects millions globally, with an estimated prevalence of 1.5 million patients in North America [1,2]. Emerging evidence suggests that chronic inflammation, a hallmark of IBD, accelerates atherosclerosis and promotes endothelial dysfunction, thereby increasing the risk of ischemic heart disease [3]. However, the exact interplay between IBD and cardiovascular disease (CVD) remains incompletely understood, with conflicting data as to whether IBD independently elevates cardiovascular risk or interacts with traditional risk factors [3,4].

Percutaneous coronary intervention (PCI) is a cornerstone therapeutic approach for managing acute coronary syndromes and obstructive coronary artery disease. While substantial progress has been made in reducing periprocedural complications, there is limited information regarding unique challenges faced by patients with systemic inflammatory diseases like IBD [5,6]. These patients may have an atypical risk profile for CVD, characterized by fewer traditional risk factors, higher rates of systemic inflammation and vascular vulnerability, and distinct post-PCI complications such as increased bleeding, in-stent restenosis (ISR), and thrombotic events [7].

This study leverages data from the National Readmission Database (NRD), one of the most comprehensive all-payer inpatient datasets in the United States, to delineate the unique cardiovascular risk profile of IBD patients compared to non-IBD controls undergoing PCI, with a focus on identifying key factors associated with post-procedural complications, mortality, and readmission.

## 2. Methods

### 2.1. Study Population

We used the United States NRD, a database managed by the Healthcare Cost and Utilization Project and run by the Agency for Healthcare Research and Quality, to analyze data from 2016 through 2020 [8]. The NRD is funded by contributions from all states affiliated with the Healthcare Cost and Utilization Project to collect information on >7 million hospitalizations yearly. Patients are tracked using verified linkage numbers within a state for a given calendar year, so the maximum follow-up period in the database is from January through November each year. Since the NRD utilizes publicly accessible, anonymized, and de-identified information, this research was exempt from requiring informed consent from participants or institutional review board approval. The NRD data were queried using claims codes from the International Classification of Diseases (ICD), Clinical Modification (ICD-10-CM). Patients 18 years of age and older who underwent PCI were identified, and this cohort was then divided into two groups: patients with and without IBD. The ICD-10 codes generated are detailed in Supplemental Table S1.

### 2.2. Study Characteristics and Outcomes

IBD encompassed both CD (ICD-10 code K50) and UC (ICD-10 code K51). Baseline demographic characteristics, including age and gender, were utilized from the available NRD variables. Missing data for the variables age and gender were excluded. Clinical variables, including hypertension, diabetes mellitus, hyperlipidemia, obesity, smoking, atrial fibrillation, valvular heart disease, renal disease, liver disease, peripheral vascular disease, coagulopathy, old myocardial infarction (MI), old PCI, chronic heart failure (CHF), and coronary artery disease (CAD) were incorporated into the analysis using the corresponding ICD-10 codes, as detailed in Supplemental Table S1.

The primary study outcome was all-cause in-hospital mortality, and secondary outcomes were cardiovascular and gastrointestinal (GI) complications. Major adverse cardiac events (MACE) were defined as cardiovascular mortality, acute heart failure, and acute stroke. Cardiovascular mortality was defined as patients who died because of cardiac arrest, cardiac arrhythmias, or heart failure. Cardiac complications included cardiac tamponade, spontaneous coronary artery dissection (SCAD), and pericardial effusion.

### 2.3. Statistical Analysis

Means with standard deviations and the number of individuals with the corresponding proportions were calculated for continuous and categorical variables, respectively. The characteristics of PCI for patients with and without IBD were compared using a survey-adjusted Wald t-test or Wilcoxon Rank-Sum test for non-normal distribution, with normality assessed by the Kolmogorov–Smirnov test, for continuous variables, and chi-square test for categorical variables. Multivariable logistic regression analysis was performed to identify the risk factors for inpatient mortality and in-hospital complications in the overall cohort of PCI-undergoing patients with IBD. To adjust for confounders, propensity score matching was used to estimate the likelihood of the development of outcomes among patients with IBD undergoing PCI. The propensity score was estimated using logistic regression based on age, female sex, CHF, diabetes mellitus, hypertension, hyperlipidemia, obesity, smoking, and atrial fibrillation. The propensity score analysis was performed in a 1:1 fashion to match 7055 patients with IBD with 7055 control patients without IBD. Matching was performed without, and balance between matched groups was assessed using standardized mean differences and empirical cumulative distribution function statistics to ensure comparability of covariates within each cohort. After matching, all standardized mean differences for the covariates were below 0.1, indicating adequate balance, and it was visualized using a covariate balance plot (Supplementary Figure S1). No caliper restrictions were applied, and treated units were matched to their nearest control unit. Unmatched control units were excluded from the analysis. A *p*-value of <0.05 was considered statistically significant. The Bonferroni correction method was used to address the challenge of multiple comparisons and to control potential Type I error rates. This correction entails adjusting the significance levels (*p*-values) for multiple comparisons. The designated overall significance level, typically set at 0.05, was divided by the number of comparisons, yielding a corrected alpha error threshold of 0.004. The adjusted significance level was compared with the calculated *p*-value for each comparison. Statistical significance, post-Bonferroni correction, was established if the adjusted *p*-value was less than or equal to the new significance level (*p* < 0.004). The analysis was conducted using Stata 17 statistical software (StataCorp, College Station, TX, USA). All analyses included sample weights, strata, and clusters to account for the complex survey design. Propensity-matching analysis was performed using R software (version 4.3.1) MatchIt package (R Foundation for Statistical Computing, Vienna, Austria). This study was conducted and reported in accordance with the Strengthening the Reporting of Observational Studies in Epidemiology (STROBE) cohort reporting guidelines.

## 3. Results

### 3.1. Baseline Demographic and Clinical Characteristics

From 2016 to 2020, a total of 2,249,868 patients who were 18 years of age and older underwent percutaneous coronary intervention (PCI). Among these, 11,499 patients (0.51%) had a concurrent diagnosis of IBD (UC: 5352; CD: 6147), with 35.4% being females (Table 1). Compared to non-IBD patients, those with IBD were slightly more likely to be female (35.4% vs. 32.6%, *p* < 0.001), smokers (52.2% vs. 51.8%, *p* < 0.001), and have a history of liver disease (5.3% vs. 3.4%, *p* < 0.001), coagulopathy (5.5% vs. 4.5%, *p* = 0.005), chronic renal failure (21.5% vs. 20.6%, *p* = 0.036), and peripheral vascular disease (13.2% vs. 12.2%, *p* = 0.03). Conversely, IBD patients had lower rates of obesity (18.4% vs. 21.7%, *p* < 0.001), diabetes (34.9% vs. 40.9%, *p* < 0.001), and hyperlipidemia (66.5% vs. 69.5%, *p* < 0.001).

In terms of cardiac comorbidities, IBD patients were more likely to have atrial fibrillation (18.3% vs. 16.7%, *p* < 0.001) and valvular heart disease (14.5% vs. 13.3%, *p* = 0.015) but were less likely to have hypertension (79.3% vs. 82.3%, *p* < 0.001). No significant differences were observed between IBD and non-IBD patients regarding CAD, CHF, history of MI, or prior PCI (*p* > 0.05). Baseline characteristics are detailed in Table 1.

### 3.2. Clinical Indications and Complications of PCI in Patients with IBD

The most frequent indication for PCI was non-ST-elevation myocardial infarction (NSTEMI), followed by ST-elevation myocardial infarction (STEMI) and cardiogenic shock, irrespective of IBD status. Patients with IBD undergoing PCI had slightly higher rates of NSTEMI (43.2% vs. 41.5%, *p* = 0.04) compared to non-IBD patients. However, no statistical differences were observed in the incidence of STEMI (29.6% vs. 30.2%, *p* = 0.16), cardiogenic shock (6.0% vs. 5.8%, *p* = 0.43), arrhythmia (4.3% vs. 3.8%, *p* = 0.23), or angina (0.18% vs. 0.19%, *p* = 0.70). The rates of PCI associated with MI complications (hemopericardium, atrial septal defect, ventricular septal defect, cardiac wall rupture without hemopericardium, rupture of chordae tendineae, papillary muscle rupture, atrial, atrial appendage, and ventricle thrombus) were also similar between the groups (0.58% vs. 0.65%, *p* = 0.78) (Table 2 part a).

Cardiovascular complications following PCI were less frequent in IBD patients, with slightly lower rates of stroke (2.0% vs. 2.8%, *p* = 0.006), acute heart failure (17.1% vs. 18.7%, *p* = 0.002), and MACE (19.9% vs. 21.7%, *p* = 0.005). However, IBD patients were more likely to experience pleural effusion (1.9% vs. 1.3%, *p* < 0.001) and cardiac complications such as tamponade, SCAD, or pericardial effusion (2.8% vs. 1.9%, *p* < 0.001). No significant differences were observed in vascular complications, including venous thromboembolism (VTE) and aortic dissection (Table 2 part b).

IBD patients had significantly higher rates of GI complications post-PCI, including acute liver failure (1.8% vs. 1.4%, *p* < 0.001), and mesenteric ischemia (0.24% vs. 0.06%, *p* < 0.001). They were also more likely to require post-catheterization blood transfusions (4.8% vs. 2.6%, *p* < 0.001). Other GI complications, such as pancreatitis, appendicitis, and cholecystitis, showed no significant differences between the two groups (Table 2 part b).

Despite these differences, all-cause mortality was comparable between IBD and non-IBD patients undergoing PCI (3.01% vs. 3.13%, *p* = 0.708). A summary of the clinical indications and complications of PCI is provided in Table 2 part b.

### 3.3. Adjusted Analysis of Complications and Mortality

Multivariable logistic regression revealed no significant difference in all-cause mortality between IBD and non-IBD patients undergoing PCI, including UC and CD subgroups. However, IBD patients had significantly higher odds of pleural effusion (OR 1.49, 95% CI [1.23–1.80], *p* < 0.001), acute liver failure (OR 1.24, 95% CI [1.006–1.535], *p* = 0.044), mesenteric ischemia (OR 3.49, 95% CI [2.05–5.93], *p* < 0.001), and cardiac complications, including tamponade, SCAD, and pericardial effusion (OR 1.38, 95% CI [1.15–1.66], *p* < 0.001). Additionally, IBD patients had nearly double the odds of requiring blood transfusions (OR 1.92, 95% CI [1.67–2.22], *p* < 0.001). In contrast, IBD was associated with a lower odds of acute heart failure (OR 0.91, 95% CI [0.84–0.98], *p* = 0.016), stroke (OR 0.81, 95% CI [0.68–0.96], *p* = 0.015), and MACE (OR 0.91, 95% CI [0.84–0.97], *p* = 0.009). No statistically significant difference in the risk of VTE, aortic dissection, and other GI complications was observed between the two groups.

A propensity-score-matching analysis was performed using a 1:1 approach to match 7055 patients with IBD to 7055 controls without IBD, to calculate the odds ratio for cardiovascular and GI complications and mortality after a PCI procedure amongst patients with IBD and taking no IBD as the reference. There was a higher rate of pleural effusion (OR 1.64, 95% CI [1.24–21.6], *p* < 0.001), cardiac complications (OR 1.31, 95% CI [1.05–1.64], *p* = 0.017), acute liver failure (OR 1.48, 95% CI [1.13–1.93], *p* = 0.004), mesenteric ischemia (OR 5.34, 95% CI [1.56–18.40], *p* = 0.007), and need for blood transfusion (OR 1.74, 95% CI [1.46–2.08], *p* < 0.001). Acute heart failure (OR 0.95, 95% CI [0.88–1.00], *p* = 0.313), stroke (OR 1.01, 95% CI [0.81–1.24], *p* = 0.957), and MACE (OR 0.95, 95% CI [0.87–1.03], *p* = 0.222) did not reach statistical significance after propensity matching. The covariate balance of propensity-matched outcomes for patients undergoing PCI with concurrent IBD is shown in Supplemental Figure S1. Table 3 and Table 4 describe the summary of the multivariable logistic regression and propensity-matched analysis results of outcomes and complications, respectively. The central illustration summarizes the methodology and main findings of our study.

### 3.4. 30-Day Cumulative Incidence of Readmission

Among IBD patients who underwent PCI, the cumulative incidence of 30-day readmission was higher when compared to the non-IBD cohort (13% vs. 10%, *p* < 0.001) with a hazard ratio (HR) 1.29 (1.21–1.38), *p* < 0.001. When IBD was sub-grouped to Crohn’s disease and ulcerative colitis, the 30-day cumulative incidence of readmission was higher for Crohn’s disease as compared to ulcerative colitis (13% vs. 12%, *p* < 0.001).

Among IBD patients who underwent PCI and experienced a VTE adverse event, the 30-day readmission cumulative incidence was significantly higher at 19% (8.2%, 33%), *p* < 0.001, compared to 17% (16%, 18%), *p* < 0.001 for non-IBD patients. However, the HR did not achieve statistical significance (HR 2.01, 95% CI 1.04–4.08, *p* = 0.052). The 30-day cumulative incidence for other adverse events is presented in Supplemental Tables S2 and S3.

### 3.5. 30-Day Readmission Events

Of the 2,249,868 patients undergoing PCI, 255,403 (11.3%) were readmitted within 30 days. Compared to non-IBD patients, those with IBD had significantly lower odds of 30-day inpatient events of acute heart failure (27.9% vs. 19.7%, *p* < 0.001; OR 0.63, 95% CI [0.53–0.76], *p* < 0.001), while the crude analysis also showed a significantly lower likelihood of cardiogenic shock (3.0% vs. 1.9%, *p* = 0.035). No significant differences were observed regarding STEMI, NSTEMI, arrhythmia, stroke, SCAD, or cardiac tamponade (Table 5 and Table 6).

## 4. Discussion

This study used the Nationwide Readmission Database to investigate a cohort of 2,249,868 patients 18 years of age and older undergoing PCI between 2016 and 2020, of whom 11,499 (0.51%) had concurrent IBD. Patients with IBD were numerically slightly more likely to be females and present with smoking history, liver disease, coagulopathy, renal failure, and peripheral vascular disease. In addition, IBD patients had a lower prevalence of traditional cardiovascular risk factors, including obesity, diabetes, and hyperlipidemia.

The prevalence of conventional CAD risk factors such as hypertension, diabetes, hyperlipidemia, and smoking has varied widely across different studies. A national dataset analysis by Kobo et al. [6] and retrospective cohort studies by Aggarwal et al. [5] and Yarur et al. [9] reported lower prevalence of traditional cardiovascular risk factors in individuals with IBD compared to matched non-IBD controls, a trend similarly observed in our study. In contrast, a United Kingdom national database analysis by Osterman et al. [10] observed a significantly higher prevalence of these risk factors in patients with IBD, specifically UC. Meanwhile, a case–control study by Sappati Biyanni et al. found no substantial differences in traditional CAD risk factors between IBD and non-IBD groups [11].

These findings suggest that traditional risk factors may not fully explain the cardiovascular risk in IBD patients, supporting the theory that chronic inflammation plays a critical role in the pathogenesis of atherosclerosis. Additionally, we observed higher rates of certain comorbidities, including atrial fibrillation and valvular heart disease, in IBD patients, which may further contribute to their distinct cardiovascular risk profile.

We analyzed post-PCI complications using both multivariable logistic regression and propensity-matched analyses. IBD patients had increased risks of pleural effusion and cardiac complications, including tamponade, SCAD, and pericardial effusion on both analyses. IBD patients also had higher rates of post-PCI mesenteric ischemia, acute liver failure, and a greater need for blood transfusions on both analyses. Despite these complications, mortality rates were comparable between the two groups.

While inflammation is hypothesized as a key driver of adverse cardiovascular outcomes in autoimmune diseases, the impact on post-PCI outcomes varies. Although our multivariable analysis supports the notion that IBD patients are less likely to develop MACE, stroke, and acute heart failure, it did not hold true after propensity score matching. In contrast, Marcusohn et al. found that patients with chronic inflammatory diseases, including IBD, had higher long-term adverse cardiovascular outcomes post-PCI, including increased readmissions for acute coronary syndrome and heart failure [12]. Likewise, Kristensen et al. highlighted that IBD was associated with worsened outcomes following MI, with increased risks of recurrent MI and cardiovascular death [13]. This parallels findings in other autoimmune diseases, such as SLE [14,15] and RA [16,17], where elevated inflammation correlates with higher risks of restenosis, recurrent revascularizations, and mortality [18,19]. Aggarwal et al. reported no difference in post-PCI major adverse cardiovascular outcomes (defined as all-cause death, MI, cerebrovascular events, and target lesion revascularization) between 131 IBD patients with confirmed coronary artery disease and 524 matched non-IBD controls [5]. The contrasting findings in our results align with several important considerations. It could be explained by the lower prevalence of conventional cardiovascular risk factors [5]. Further, IBD patients tend to be diagnosed with CAD at a younger age compared to non-IBD patients, which may contribute to better outcomes post-PCI [20]. This selection bias toward a healthier IBD phenotype may explain the absence of worse outcomes despite their chronic inflammatory burden. Certain anti-inflammatory treatments used in IBD, such as thiopurines, have been associated with a decreased incidence of cardiovascular events. This suggests that effective management of inflammation in IBD may mitigate some cardiovascular risks [20].

Our multivariable analysis revealed that IBD patients had a significantly increased risk of GI and hepatic complications, including acute liver failure, mesenteric ischemia, and a nearly twofold increased likelihood of requiring blood transfusions post-PCI. This aligns with the findings of Kobo et al. [6] who reported elevated bleeding risks and Antia et al. [21] who reported a 66% higher rate of blood product transfusion among IBD patients. The increased GI complications could reflect the heightened vulnerability of the GI tract due to the underlying inflammatory state and use of antiplatelet or anticoagulant therapy during PCI. Given the elevated bleeding risks and potential for adverse long-term outcomes, these findings underscore the importance of balancing inflammation control and antithrombotic management in IBD patients undergoing PCI. 

Despite these risks, our study suggests that IBD patients may experience fewer complications, such as MACE, which did not hold true after propensity matching, and that there is no significant mortality difference. Aggarwal et al. [5] reported a nearly two-fold lower risk of MACE and death in IBD patients compared to non-IBD controls, though these findings were not statistically significant. In contrast, Kobo et al. [6] reported lower vascular complications and mortality in IBD patients post-PCI, similar to Antia et al. [21] who noted lower rates of mortality and cardiac arrest among IBD patients undergoing PCI despite higher resource utilization and prolonged hospital stay in a nationwide analysis.

Inflammation may play a key role in adverse cardiovascular outcomes in IBD patients [22]. Mami et al. demonstrated that IBD-driven systemic inflammation worsens MI severity and disrupts post-ischemic cardiac repair [23]. Elevated pro-inflammatory cytokines, such as IL-6 and TNF-α, contribute to endothelial dysfunction, platelet activation, and thrombosis, promoting atherosclerosis and adverse post-PCI remodeling [24,25]. IL-6, in particular, is linked to higher risks of restenosis, adverse cardiovascular events, and mortality [26,27,28]. Reduced mannose-binding lectin levels in IBD further impair endothelial repair, increasing susceptibility to in-stent restenosis [29,30].

Anti-inflammatory therapies, such as 5-ASA compounds, may offer cardioprotective effects. A Danish cohort study found a significantly lower risk of ischemic heart disease in IBD patients using 5-ASA, highlighting the potential benefits of inflammation modulation [31]. Furthermore, gut microbiota dysbiosis in IBD exacerbates systemic inflammation, with microbial products entering the circulation and triggering inflammatory pathways that contribute to endothelial dysfunction and arterial stiffness [32,33]. This gut-heart axis may explain the elevated thrombotic and GI complications observed post-PCI in IBD patients, and the interplay between chronic inflammation, endothelial dysfunction, and GI vulnerability underscores the importance of tailored management strategies.

Our findings have several important implications for clinical practice. First, IBD should be recognized as a cardiovascular risk-enhancing factor when assessing patients for atherosclerotic cardiovascular disease (ASCVD) risk, as current American College of Cardiology/American Heart Association guidelines acknowledge that chronic inflammatory conditions favor statin therapy in patients at borderline or intermediate risk [34]. Routine screening for traditional cardiovascular risk factors, including hypertension, diabetes, and dyslipidemia, should be performed in all IBD patients with aggressive management, recognizing that standard ASCVD risk calculators may underestimate actual risk, particularly in younger individuals [34]. Second, the most critical prophylactic strategy is aggressive control of intestinal inflammation, as disease activity correlates with cardiovascular risk and anti-TNF biologics appear protective against cardiovascular events, while prolonged corticosteroid exposure correlates with adverse vascular outcomes [34,35]. Third, the observed increased bleeding complications alongside cardiac complications in IBD patients undergoing PCI create a challenging therapeutic dilemma requiring individualized antithrombotic strategies. Prior studies confirm that IBD patients undergoing PCI have 35–42% increased odds of major bleeding, necessitating consideration of shortened dual antiplatelet therapy duration, routine proton pump inhibitor co-prescription to reduce GI bleeding risk, and close multidisciplinary coordination between gastroenterology and cardiology to balance antiplatelet intensity against bleeding risk [6]. Finally, the substantially elevated risk of mesenteric ischemia (OR 5.34) warrants heightened clinical suspicion for this complication in the peri-procedural period. These findings underscore the need for integrated cardiovascular-gastroenterological care pathways for IBD patients requiring coronary intervention.

## 5. Limitations

This study has some potential limitations. First, as a retrospective analysis utilizing the NRD, our findings are inherently subject to the constraints of administrative data, including potential coding inaccuracies, misclassification bias, and the absence of granular clinical details such as disease activity and specific treatment regimens. Second, the lack of long-term longitudinal follow-up limits the ability to assess long-term outcomes such as in-stent restenosis or late mortality, which are crucial for understanding the full cardiovascular risk spectrum in IBD patients post-PCI. Third, although propensity score matching was employed to minimize confounding, residual confounding by unmeasured variables such as age, socioeconomic status, physical activity, and adherence to therapy remains a possibility. Furthermore, the absence of procedural details, such as stent type, lesion complexity, and peri-procedural pharmacotherapy, limits the evaluation of factors that may influence post-PCI outcomes. Additionally, hospitalized IBD patients undergoing PCI likely represent a sicker subset, potentially limiting the generalizability of our findings to the broader IBD population. Finally, given the NRD’s focus on U.S. hospitalizations, the applicability of our results to other populations with different healthcare systems, genetic backgrounds, and environmental exposures may be limited.

## 6. Conclusions

Our NRD-based study highlights the unique cardiovascular risk profiles and clinical outcomes of IBD patients undergoing PCI. Despite lower rates of traditional risk factors like diabetes and hyperlipidemia, IBD patients faced elevated risks of pleural effusion, GI complications, and the need for blood transfusions while showing a lower likelihood of MACE, HF, and stroke and no difference in post-PCI mortality. These findings emphasize the critical role of systemic inflammation in shaping post-PCI outcomes and underscore the need for tailored management strategies. Future research should focus on the long-term impact of biological therapies and microbiota-targeted interventions to guide evidence-based care and optimize outcomes in this high-risk population.

## Figures and Tables

**Table 1:** Baseline characteristics of patients with and without inflammatory bowel disease undergoing percutaneous coronary intervention.

Variables	Without IBD N = 2,238,370, n (%)	with CD N = 6147, n (%)	with UC N = 5352, n (%)	Total IBD N = 11,499, n (%)	*p*-Value
Female	729,700 (32.6)	2429 (39.51)	1645 (30.73)	4074 (35.43)	<0.001
Age (in years)					<0.001
18–44	109,009 (4.87)	317 (5.16)	155 (2.90)	472 (4.11)	
45–64	932,826 (41.67)	2521 (41.01)	2053 (38.36)	4574 (39.77)	
65–84	1,053,168 (47.05)	3001 (48.83)	2830 (52.88)	5831 (50.71)	
>84	143,366 (6.40)	308 (5.01)	314 (5.87)	622 (5.41)	
Chronic heart failure	259,226 (11.58)	742 (12.08)	587 (10.97)	1330 (11.56)	0.469
Obesity	484,828 (21.66)	1127 (18.34)	993 (18.56)	2121 (18.44)	<0.001
Smoking	1,159,836 (51.82)	3462 (56.31)	2542 (47.49)	6003 (52.21)	<0.001
Atrial fibrillation	375,384 (16.77)	1050 (17.08)	1057 (19.75)	2107 (18.33)	<0.001
Hyperlipidemia	1,555,096 (69.47)	4024 (65.46)	3621 (67.67)	7646 (66.49)	<0.001
Hypertension	1,841,027 (82.25)	4918 (80.01)	4201 (78.50)	9120 (79.31)	<0.001
Diabetes mellitus	915,581 (40.90)	2179 (35.45)	1831 (34.21)	4010 (34.87)	<0.001
Coronary artery disease	2,066,458 (92.32)	5657 (92.03)	4880 (91.18)	10,536 (91.63)	0.088
Valvular heart disease	298,449 (13.33)	859 (13.98)	813 (15.18)	1672 (14.54)	0.015
Renal failure	461,084 (20.60)	1385 (22.54)	1088 (20.34)	2474 (21.51)	0.036
Liver disease	76,882 (3.43)	311 (5.05)	300 (5.60)	610 (5.31)	<0.001
Peripheral vascular disease	272,272 (12.16)	847 (13.77)	675 (12.62)	1522 (13.24)	0.030
Coagulopathy	101,890 (4.55)	328 (5.34)	300 (5.60)	628 (5.46)	0.005
History of myocardial infarction	411,300 (18.37)	1215 (19.77)	981 (18.33)	2196 (19.10)	0.161
History of percutaneous coronary intervention	37,745 (1.69)	120 (1.96)	103 (1.92)	223 (1.94)	0.355

Abbreviations: IBD: Inflammatory bowel disease; CD: Crohn’s disease; UC: Ulcerative colitis.

**Table 2:** **a:** Indications for percutaneous coronary intervention in inflammatory bowel disease. **b:** Cardiovascular and Gastrointestinal Complications Post Percutaneous Coronary Intervention.

**a**					
	**Without IBD** **N = 2,238,370, n (%)**	**with CD** **N = 6147, n (%)**	**with UC** **N = 5352, n (%)**	**Total IBD** **N = 11,499, n (%)**	***p*-Value**
Unstable Angina	4211 (0.19)	14 (0.23)	7.3 (0.14)	21 (0.18)	0.704
STEMI	674,833 (30.15)	1757 (28.58)	1644 (30.71)	3400 (29.57)	0.158
NSTEMI	929,863 (41.54)	2678 (43.56)	2294 (42.86)	4972 (43.23)	0.039
Arrhythmia	85,501(3.82)	259 (4.21)	232 (4.34)	491 (4.27)	0.230
Cardiogenic shock	129,054 (5.77)	354 (5.75)	341 (6.37)	694 (6.04)	0.427
MI complications	14,638 (0.65)	35 (0.58)	31 (0.58)	66 (0.58)	0.781
**b**					
	**Without IBD** **N = 2,238,370, n (%)**	**with CD** **N = 6147, n (%)**	**with UC** **N = 5352, n (%)**	**Total IBD** **N = 11,499, n (%)**	***p*** **-value**
**Cardiovascular complications**					
Aortic dissection	38,205 (1.71)	126 (2.05)	115 (2.15)	241 (2.10)	0.076
Cardiac complications (tamponade, SCAD, and pericardial effusion)	43,137 (1.93)	178 (2.89)	139 (2.60)	317 (2.75)	<0.001
MACE	486,002 (21.71)	1218 (19.81)	1071 (20.02)	2289 (19.91)	0.005
Acute heart failure	419,156 (18.73)	1025 (16.68)	940 (17.56)	1965 (17.09)	0.002
Stroke	63,620 (2.84)	153 (2.50)	107 (2.00)	260 (2.26)	0.006
Pleural effusion	29,249 (1.31)	114 (1.85)	110 (2.06)	224 (1.94)	<0.001
VTE	8203 (0.37)	29 (0.47)	30 (0.57)	60 (0.52)	0.123
**Gastrointestinal complications**					
Acute liver failure	31,988 (1.43)	82 (1.34)	128 (2.38)	210 (1.83)	<0.001
Mesenteric ischemia	1462 (0.065)	14 (0.22)	14 (0.26)	28 (0.24)	<0.001
PRBC Transfusion	57,958 (2.59)	248 (4.04)	306 (5.72)	554 (4.82)	<0.001
Others (pancreatitis, cholecystitis)	8818 (0.39)	34 (0.55)	21 (0.40)	55 (0.48)	0.394
**All-cause Mortality**					
Mortality	67,251 (3.01)	201 (3.27)	159 (2.98)	360 (3.13)	0.708

Abbreviations: STEMI: ST-elevation myocardial infarction; NSTEMI: non-ST elevation myocardial infarction; MI: Myocardial infarction; IBD: Inflammatory bowel disease; CD: Crohn’s disease; UC: Ulcerative colitis; SCAD: Spontaneous coronary artery dissection; VTE: Venous thromboembolism; MACE: Major adverse cardiac events; PRBC: Packed red blood cells.

**Table 3:** Multivariable logistic regression analysis of all the outcomes and complications.

Outcomes		Odds Ratio (95% Confidence Interval)	*p*-Value
Mortality	CD	1.08 (0.87–1.34)	0.448
	UC	0.93 (0.74–1.17)	0.564
	IBD	1.01 (0.86–1.18)	0.855
Venous thromboembolism	CD	1.28 (0.76–2.17)	0.349
	UC	1.50 (0.94–2.39)	0.083
	IBD	1.39 (0.98–1.96)	0.064
Pleural effusion	CD	1.43 (1.09–1.89)	0.009
	UC	1.55 (1.19–2.00)	0.001
	IBD	1.49 (1.23–1.80)	<0.001
Aortic dissection	CD	1.20 (0.92–1.57)	0.169
	UC	1.19 (0.91–1.54)	0.187
	IBD	1.19 (0.99–1.43)	0.053
Cardiac complications (tamponade, SCAD, and pericardial effusion)	CD	1.43 (1.14–1.80)	0.002
	UC	1.33 (0.99–1.77)	0.053
	IBD	1.38 (1.15–1.66)	<0.001
Major adverse cardiac events	CD	0.92 (0.83–1.01)	0.092
	UC	0.90 (0.81–0.99)	0.038
	IBD	0.91 (0.84–0.97)	0.009
Acute heart failure	CD	0.90 (0.81–1.00)	0.051
	UC	0.92 (0.83–1.02)	0.141
	IBD	0.91 (0.84–0.98)	0.016
Stroke	CD	0.89 (0.71–1.12)	0.344
	UC	0.71 (0.55–0.92)	0.010
	IBD	0.81 (0.68–0.96)	0.015
Acute liver failure	CD	0.92 (0.66–1.27)	0.625
	UC	1.60 (1.21–2.11)	0.001
	IBD	1.24 (1.00–1.53)	0.044
Mesenteric ischemia	CD	3.22 (1.66–6.24)	0.001
	UC	3.80 (1.68–8.60)	0.001
	IBD	3.49 (2.05–5.93)	<0.001
Packed red blood cell transfusion	CD	1.59 (1.33–1.90)	<0.001
	UC	2.32 (1.86–2.90)	<0.001
	IBD	1.92 (1.67–2.22)	<0.001
Gastrointestinal complications (pancreatitis and cholecystitis)	CD	1.41 (0.87–2.30)	0.160
	UC	1.02 (0.57–1.82)	0.937
	IBD	1.23 (0.84–1.79)	0.272

Abbreviations: CD: Crohn’s disease; UC: Ulcerative colitis; IBD: Inflammatory bowel disease; SCAD: Spontaneous coronary artery dissection.

**Table 4:** Propensity-matched results of all the outcomes and complications.

Outcomes	Odds Ratio (95% Confidence Interval)	*p*-Value	Statistical Significance After Bonferroni Correction (*p* < 0.004)
Mortality	0.86 (0.72–1.04)	0.113	No
Venous thromboembolism	1.16 (0.71–1.86)	0.548	No
Pleural effusion	1.64 (1.24–2.16)	<0.001	Yes
Aortic dissection	1.11(0.87–1.41)	0.387	No
Cardiac complications	1.31 (1.05–1.64)	0.017	No
Major adverse cardiac events	0.95 (0.87–1.03)	0.222	No
Acute heart failure	0.95 (0.88–1.00)	0.313	No
Stroke	1.01 (0.81–1.24)	0.957	No
Acute liver failure	1.48 (1.13–1.93)	0.004	No
Mesenteric ischemia	5.34 (1.56–18.40)	0.007	No
Packed red blood cell transfusion	1.74 (1.46–2.08)	<0.001	Yes
Gastrointestinal complications (pancreatitis and cholecystitis)	1.56 (0.94–2.59)	0.082	No

**Table 5:** Number of 30-day readmissions after percutaneous coronary intervention.

	Without IBD N = 253,679, n (%)	with IBD N = 1725, n (%)	*p*-Value
STEMI	34,876 (13.75)	213 (12.36)	0.273
NSTEMI	52,839 (20.83)	340 (19.74)	0.442
Arrhythmia	3511 (1.38)	27 (1.57)	0.672
Cardiogenic shock	7560 (2.98)	32 (1.86)	0.035
Stroke	13,976 (5.51)	96 (5.54)	0.970
Acute heart failure	70,643 (27.85)	339 (19.66)	<0.001
SCAD	959 (0.38)	9.2 (0.53)	0.525
Cardiac tamponade	818 (0.32)	4.6 (0.26)	0.735

Abbreviations: STEMI: ST-elevation myocardial infarction; NSTEMI: non-ST elevation myocardial infarction; SCAD: Spontaneous coronary artery dissection; IBD: Inflammatory bowel disease.

**Table 6:** Odds of cardiovascular risk in 30-day readmission with inflammatory bowel disease compared to non-inflammatory bowel disease patients.

Variables	Odds Ratio (95% Confidence Interval)	*p*-Value
STEMI	0.85 (0.68–1.06)	0.167
NSTEMI	0.94 (0.79–1.12)	0.529
Arrhythmia	1.10 (0.60–2.00)	0.745
Stroke	1.03 (0.72–1.46)	0.849
Acute heart failure	0.63 (0.53–0.76)	<0.001
SCAD	1.29 (0.45–3.73)	0.490
Cardiac tamponade	0.76 (0.24–2.41)	0.646

Abbreviations: STEMI: ST-elevation myocardial infarction; NSTEMI: non-ST elevation myocardial infarction; SCAD: Spontaneous coronary artery dissection.

## Data Availability

All the data pertinent to the article is available with the corresponding author on reasonable request.

## Supplementary Materials

The following supporting information can be downloaded at: https://www.mdpi.com/article/10.3390/jcm15062431/s1, Figure S1: Covariate balance plot; Figure S2: 30-day cumulative incidence plot based on adverse events; Table S1: ICD-10 diagnostic and procedure codes; Table S2: Cumulative incidence of readmission in percentages; Table S3: Predictors of readmission.

## Abbreviations

PCI	Percutaneous coronary intervention
IBD	Inflammatory bowel disease
UC	Ulcerative colitis
CD	Crohn’s disease
NRD	National Readmission Database
NSTEMI	Non-ST-elevation myocardial infarction
STEMI	ST-elevation myocardial infarction

## References

[1] Patel R.S., Rohit Reddy S., Llukmani A., Hashim A., Haddad D.R., Patel D.S., Ahmad F., Gordon D.K. (2021). Cardiovascular Manifestations in Inflammatory Bowel Disease: A Systematic Review of the Pathogenesis and Management of Pericarditis. Cureus.

[2] Jairath V., Feagan B.G. (2020). Global Burden of Inflammatory Bowel Disease. Lancet Gastroenterol. Hepatol..

[3] Chen B., Collen L.V., Mowat C., Isaacs K.L., Singh S., Kane S.V., Farraye F.A., Snapper S., Jneid H., Lavie C.J. (2022). Inflammatory Bowel Disease and Cardiovascular Diseases. Am. J. Med..

[4] Wu H., Hu T., Hao H., Hill M.A., Xu C., Liu Z. (2022). Inflammatory Bowel Disease and Cardiovascular Diseases: A Concise Review. Eur. Heart J. Open.

[5] Aggarwal A., Atreja A., Kapadia S., Lopez R., Achkar J.-P. (2015). Conventional Risk Factors and Cardiovascular Outcomes of Patients with Inflammatory Bowel Disease with Confirmed Coronary Artery Disease. Inflamm. Bowel Dis..

[6] Kobo O., Mohamed M.O., Farmer A.D., Alraies C.M., Patel T., Sharma K., Nolan J., Bagur R., Roguin A., Mamas M.A. (2020). Outcomes of Percutaneous Coronary Intervention in Patients with Crohn’s Disease and Ulcerative Colitis (from a Nationwide Cohort). Am. J. Cardiol..

[7] Pepe M., Napoli G., Carulli E., Moscarelli M., Forleo C., Nestola P.L., Biondi-Zoccai G., Giordano A., Favale S. (2021). Autoimmune Diseases in Patients Undergoing Percutaneous Coronary Intervention: A Risk Factor for in-Stent Restenosis?. Atherosclerosis.

[8] Healthcare Cost and Utilization Project (HCUP). https://www.ahrq.gov/data/hcup/index.html.

[9] Yarur A.J., Deshpande A.R., Pechman D.M., Tamariz L., Abreu M.T., Sussman D.A. (2011). Inflammatory Bowel Disease Is Associated with an Increased Incidence of Cardiovascular Events. Am. J. Gastroenterol..

[10] Osterman M.T., Yang Y.-X., Brensinger C., Forde K.A., Lichtenstein G.R., Lewis J.D. (2011). No Increased Risk of Myocardial Infarction among Patients with Ulcerative Colitis or Crohn’s Disease. Clin. Gastroenterol. Hepatol..

[11] Sappati Biyyani R.S.R., Fahmy N.M., Baum E., Nelson K.M., King J.F. (2009). Inflammatory Bowel Disease and Coronary Artery Disease. Indian. J. Gastroenterol..

[12] Marcusohn E., Zukermann R., Kerner A., Roguin A., Kobo O. (2021). Long-Term Outcomes of Patients with Chronic Inflammatory Diseases after Percutaneous Coronary Intervention. Catheter. Cardiovasc. Interv..

[13] Kristensen S.L., Ahlehoff O., Lindhardsen J., Erichsen R., Lamberts M., Khalid U., Nielsen O.H., Torp-Pedersen C., Gislason G.H., Hansen P.R. (2014). Prognosis after First-Time Myocardial Infarction in Patients with Inflammatory Bowel Disease According to Disease Activity: Nationwide Cohort Study. Circ. Cardiovasc. Qual. Outcomes.

[14] Maksimowicz-McKinnon K., Selzer F., Manzi S., Kip K.E., Mulukutla S.R., Marroquin O.C., Smitherman T.C., Kuller L.H., Williams D.O., Wasko M.C.M. (2008). Poor 1-Year Outcomes after Percutaneous Coronary Interventions in Systemic Lupus Erythematosus: Report from the National Heart, Lung, and Blood Institute Dynamic Registry. Circ. Cardiovasc. Interv..

[15] Lai C.-H., Lai W.-W., Chiou M.-J., Lin W.-C., Yang Y.-J., Li C.-Y., Tsai L.-M. (2016). Outcomes of Percutaneous Coronary Intervention in Patients with Rheumatoid Arthritis and Systemic Lupus Erythematosus: An 11-Year Nationwide Cohort Study. Ann. Rheum. Dis..

[16] Dawson L.P., Dinh D., O’Brien J., Duffy S.J., Guymer E., Brennan A., Clark D., Oqueli E., Hiew C., Freeman M. (2021). Outcomes of Percutaneous Coronary Intervention in Patients with Rheumatoid Arthritis. Am. J. Cardiol..

[17] Wassif H., Saad M., Desai R., Hajj-Ali R.A., Menon V., Chaudhury P., Nakhla M., Puri R., Prasada S., Reed G.W. (2022). Outcomes Following Acute Coronary Syndrome in Patients with and Without Rheumatic Immune-Mediated Inflammatory Diseases. J. Am. Heart Assoc..

[18] Conrad N., Verbeke G., Molenberghs G., Goetschalckx L., Callender T., Cambridge G., Mason J.C., Rahimi K., McMurray J.J.V., Verbakel J.Y. (2022). Autoimmune Diseases and Cardiovascular Risk: A Population-Based Study on 19 Autoimmune Diseases and 12 Cardiovascular Diseases in 22 Million Individuals in the UK. Lancet.

[19] Nochioka K., Biering-Sørensen T., Hansen K.W., Sørensen R., Pedersen S., Jørgensen P.G., Iversen A., Shimokawa H., Jeger R., Kaiser C. (2017). Long-Term Outcomes in Patients with Rheumatologic Disorders Undergoing Percutaneous Coronary Intervention: A BAsel Stent Kosten-Effektivitäts Trial-PROspective Validation Examination (BASKET-PROVE) Sub-Study. Eur. Heart J. Acute Cardiovasc. Care.

[20] Thapa S.D., Hadid H., Schairer J., Imam W., Jafri S.-M. (2015). Effect of Inflammatory Bowel Disease-Related Characteristics and Treatment Interventions on Cardiovascular Disease Incidence. Am. J. Med. Sci..

[21] Antia A., Aomreore K., Udongwo N., Menon S., Ibebuogu U. (2024). In-Hospital Outcomes and Trends of Patients with Autoimmune Diseases Undergoing Percutaneous Coronary Intervention: A Nationwide Analysis. Cardiovasc. Revasc. Med..

[22] Sinh P., Cross R.K. (2024). Cardiovascular Comorbidities and Inflammatory Bowel Disease: Causes and Consequences. Gastroenterol. Hepatol..

[23] Mami W., Znaidi-Marzouki S., Doghri R., Ben Ahmed M., Znaidi S., Messadi E. (2023). Inflammatory Bowel Disease Increases the Severity of Myocardial Infarction after Acute Ischemia-Reperfusion Injury in Mice. Biomedicines.

[24] Kamperidis N., Kamperidis V., Zegkos T., Kostourou I., Nikolaidou O., Arebi N., Karvounis H. (2021). Atherosclerosis and Inflammatory Bowel Disease-Shared Pathogenesis and Implications for Treatment. Angiology.

[25] Pai J.K., Pischon T., Ma J., Manson J.E., Hankinson S.E., Joshipura K., Curhan G.C., Rifai N., Cannuscio C.C., Stampfer M.J. (2004). Inflammatory Markers and the Risk of Coronary Heart Disease in Men and Women. N. Engl. J. Med..

[26] Ito H. (2004). Novel Therapy for Crohn’s Disease Targeting IL-6 Signalling. Expert. Opin. Ther. Targets.

[27] Nevulis M.G., Baker C., Lebovics E., Frishman W.H. (2018). Overview of Link Between Inflammatory Bowel Disease and Cardiovascular Disease. Cardiol. Rev..

[28] Ridker P.M., Libby P., MacFadyen J.G., Thuren T., Ballantyne C., Fonseca F., Koenig W., Shimokawa H., Everett B.M., Glynn R.J. (2018). Modulation of the Interleukin-6 Signalling Pathway and Incidence Rates of Atherosclerotic Events and All-Cause Mortality: Analyses from the Canakinumab Anti-Inflammatory Thrombosis Outcomes Study (CANTOS). Eur. Heart J..

[29] Bagyura Z., Kiss L., Berta B., Szilágyi Á., Hirschberg K., Széplaki G., Lux Á., Szelid Z., Soós P., Merkely B. (2017). High Rate of In-Stent Restenosis after Coronary Intervention in Carriers of the Mutant Mannose-Binding Lectin Allele. BMC Cardiovasc. Disord..

[30] Saevarsdottir S., Oskarsson O.O., Aspelund T., Eiriksdottir G., Vikingsdottir T., Gudnason V., Valdimarsson H. (2005). Mannan Binding Lectin as an Adjunct to Risk Assessment for Myocardial Infarction in Individuals with Enhanced Risk. J. Exp. Med..

[31] Rungoe C., Basit S., Ranthe M.F., Wohlfahrt J., Langholz E., Jess T. (2013). Risk of Ischaemic Heart Disease in Patients with Inflammatory Bowel Disease: A Nationwide Danish Cohort Study. Gut.

[32] Nesci A., Carnuccio C., Ruggieri V., D’Alessandro A., Di Giorgio A., Santoro L., Gasbarrini A., Santoliquido A., Ponziani F.R. (2023). Gut Microbiota and Cardiovascular Disease: Evidence on the Metabolic and Inflammatory Background of a Complex Relationship. Int. J. Mol. Sci..

[33] Sardu C., Consiglia Trotta M., Santella B., D’Onofrio N., Barbieri M., Rizzo M.R., Sasso F.C., Scisciola L., Turriziani F., Torella M. (2021). Microbiota Thrombus Colonization May Influence Athero-Thrombosis in Hyperglycemic Patients with ST Segment Elevation Myocardialinfarction (STEMI). Marianella Study. Diabetes Res. Clin. Pract..

[34] Cainzos-Achirica M., Glassner K., Zawahir H.S., Dey A.K., Agrawal T., Quigley E.M.M., Abraham B.P., Acquah I., Yahya T., Mehta N.N. (2020). Inflammatory Bowel Disease and Atherosclerotic Cardiovascular Disease: JACC Review Topic of the Week. J. Am. Coll. Cardiol..

[35] Puca P., Coppola G., Parello S., Capobianco I., Colantuono S., Scaldaferri F., Papa A. (2025). Atherosclerotic Cardiovascular Disease and Inflammatory Bowel Disease: Epidemiology, Pathogenesis and Risk Assessment. Best. Pract. Res. Clin. Gastroenterol..

