# Supplementary material for: Outcomes of Percutaneous Coronary Intervention in Patients with Inflammatory Bowel Disease
Source: J Clin Med. 2026 Mar 22;15(6):2431. doi: 10.3390/jcm15062431 (PMC13028590; doi:10.3390/jcm15062431)
Supplement: Supplementary file 1 [file jcm-15-02431-s001.zip › jcm-4200776-supplementary.pdf]

Supplementary Table S1: ICD-10 diagnostic and procedure codes.

Variables	ICD-10 codes
Ulcerative colitis	K51
Crohn's disease	K50
Percutaneous coronary intervention	0270346, 0270376, 02733GZ, 02703G6, 0270356, 0271356, 02703D6, 02713Z6, 0271366, 0272366, 02713F6, 02723F6, 0272346, 0273346, 0272376, 02733G6, 0273356, 027034Z, 02733Z6, 02703GZ, 027035Z, 02713DZ, 02703ZZ, 02713ZZ, 02713FZ, 02723FZ, 027234Z, 027337Z, 02723GZ, 027335Z, 02713G6, 02733ZZ, 0272356, 02703F6, 02723Z6, 0271346, 027036Z, 02703FZ, 027134Z, 027235Z, 02723DZ, 02723ZZ, 027336Z, 02733FZ
Cardiac arrhythmias	Z450, Z950, Y821, I501, R000, R001, R008, I459, I456, I441, I442, I443, I47, I48, I49
Stroke	I60, I61, I62, I63, I650, I688, O255, O873
Chronic heart failure	I5022, I5032, I5042, I509
Acute heart failure	I501, I5020, I5021, I5023, I5030, I5031, I5033, I5040, I5041, I5043
Spontaneous coronary artery dissection	I2542
Cardiac tamponade	I314
Pericardial effusion	I313, I312
Coronary artery disease	I201, I25, I208, I209
ST-segment elevation myocardial infarction	I210, I211, I212, I213
Non ST-segment elevation myocardial infarction	I200, I214

Myocardial infarction complications	I23
Packed red blood cell transfusion	30233N, 30243P, 30233P
Aortic dissection	I71
Acute liver failure	K720, K729
Diabetes mellitus	E08, E09, E10, E11, E13
Hypertension	I1
Hyperlipidemia	E780, E781, E782, E783, E784, E785
Obesity	E660, E661, E662, E668, E669, Z683, Z684, O9921
Smoking	F17, Z720, Z87891

Figure S1: Covariate balance plot.

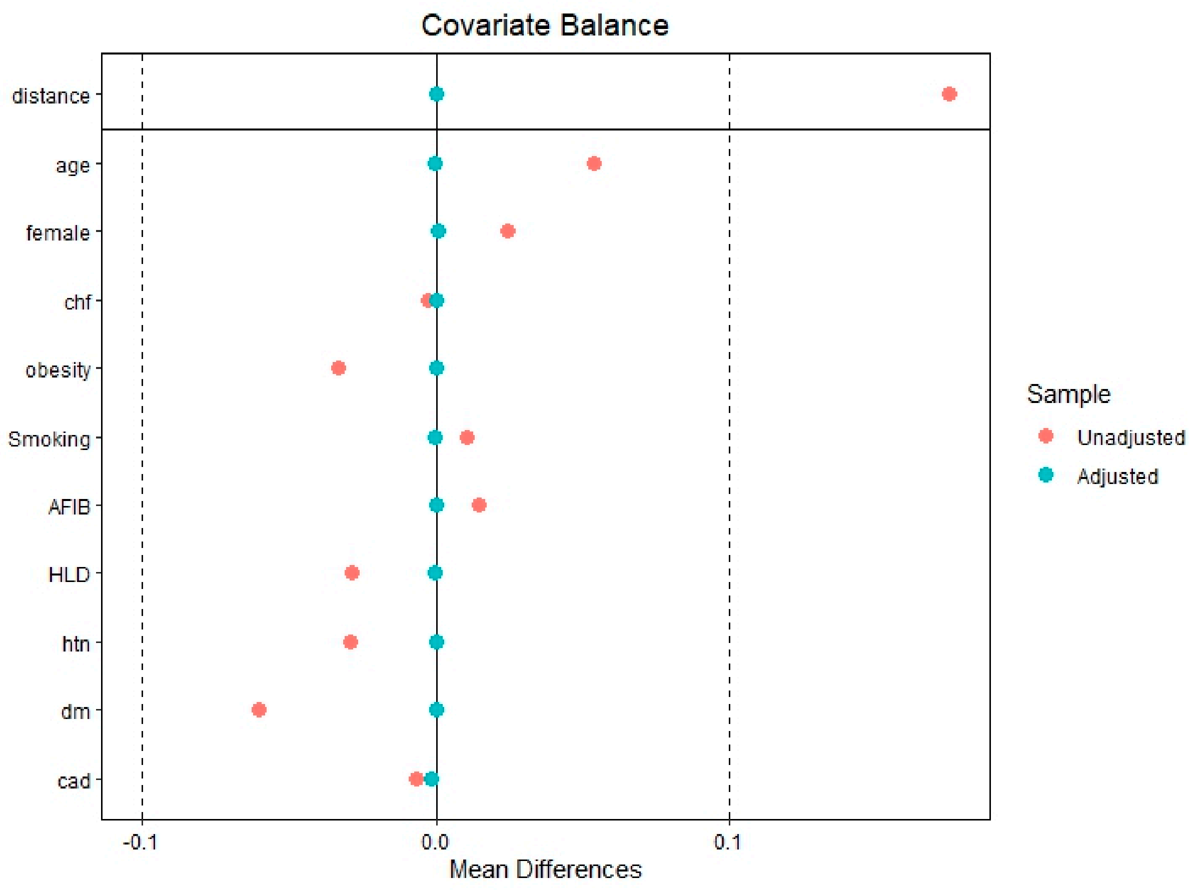

Table S2

Cumulative incidence of readmission in percentages			
	Without IBD	With IBD	P
All-cause mortality	10(10-10)	13(12-14)	<0.001
Venous thromboembolism	17(16-18)	19(8.2-33)	<0.001
Pleural effusion	19(19-20)	23(16-30)	<0.001
Aortic dissection	13(13-14)	15(9.5-21)	<0.001
Cardiac complication	13(13-13)	13(8.6-18)	<0.001
Acute heart failure	17(17-17)	19(17-21)	<0.001
Stroke	16(15-16)	19914-25)	<0.001
Acute liver failure	10(10-11)	14(9.0-21)	<0.001
Mesenteric Ischemia	11(9.3-14)	13(1.9-34)	<0.001
Transfusion	19(19-20)	25(21-30)	<0.001
Gastrointestinal Complication	16(15-17)	18(7.8-31)	<0.001

Table S3

Predictors of readmission		
	HR	P
All cause	1.29	<0.01
Venous thromboembolism	2.01	0.05
Pleural effusion	2.43	<0.01
Aortic dissection	1.60	0.01
Cardiac complication	1.36	0.12
Acute heart failure	1.98	<0.01
Stroke	2.03	<0.01
Acute liver failure	1.60	0.02
Mesenteric Ischemia	1.00	>0.9
Transfusion	2.73	<0.01
Gastrointestinal Complication	2.39	<0.005

Figure S2: Thirty-day cumulative incidence plot based on adverse events.

1. **Venous thromboembolism.**

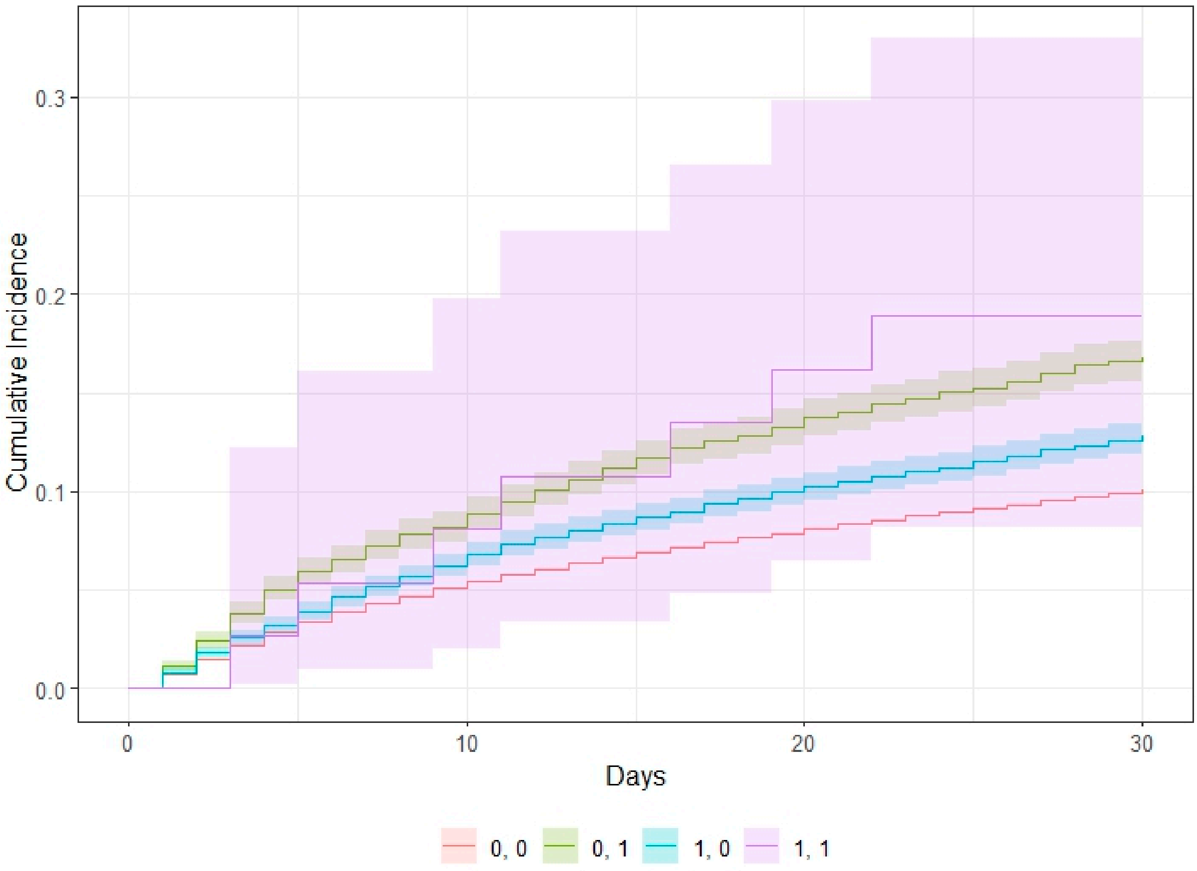

2. Pleural effusion.

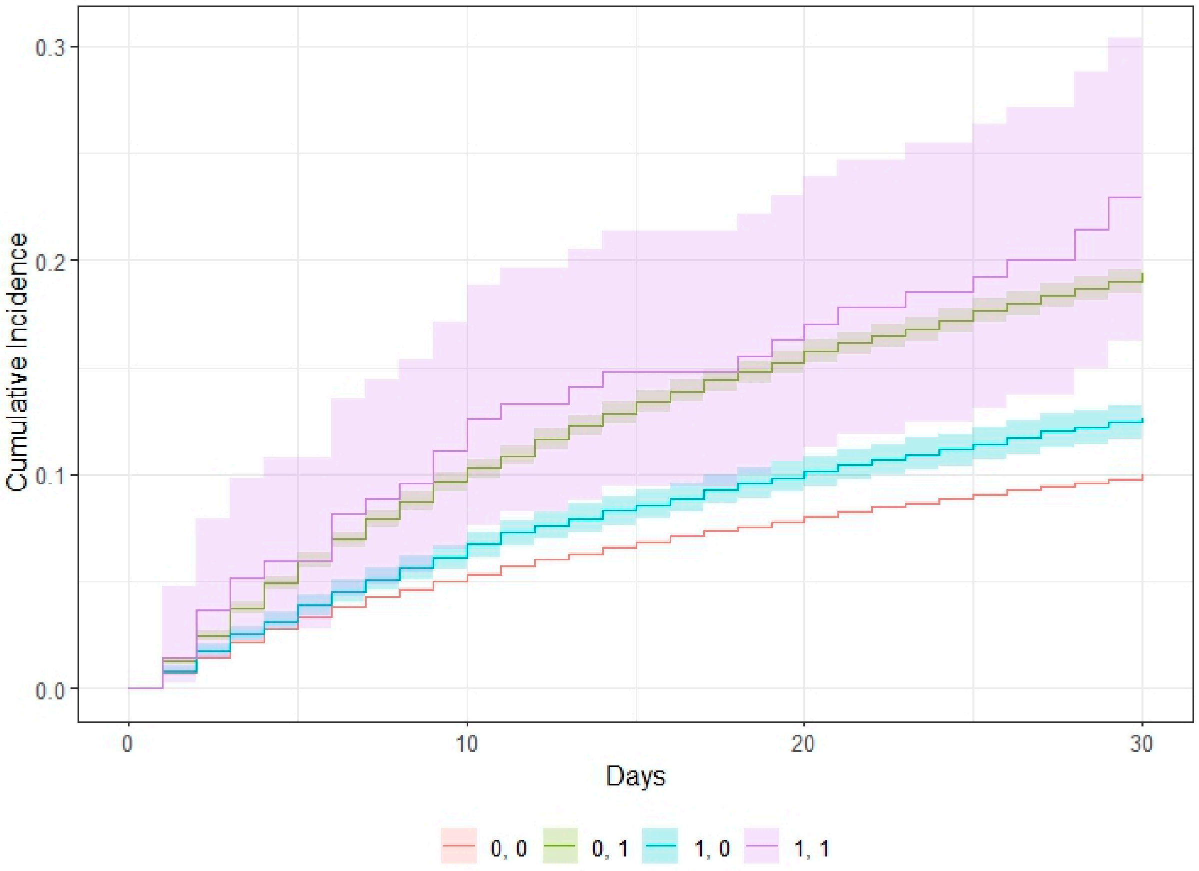

3. Aortic dissection.

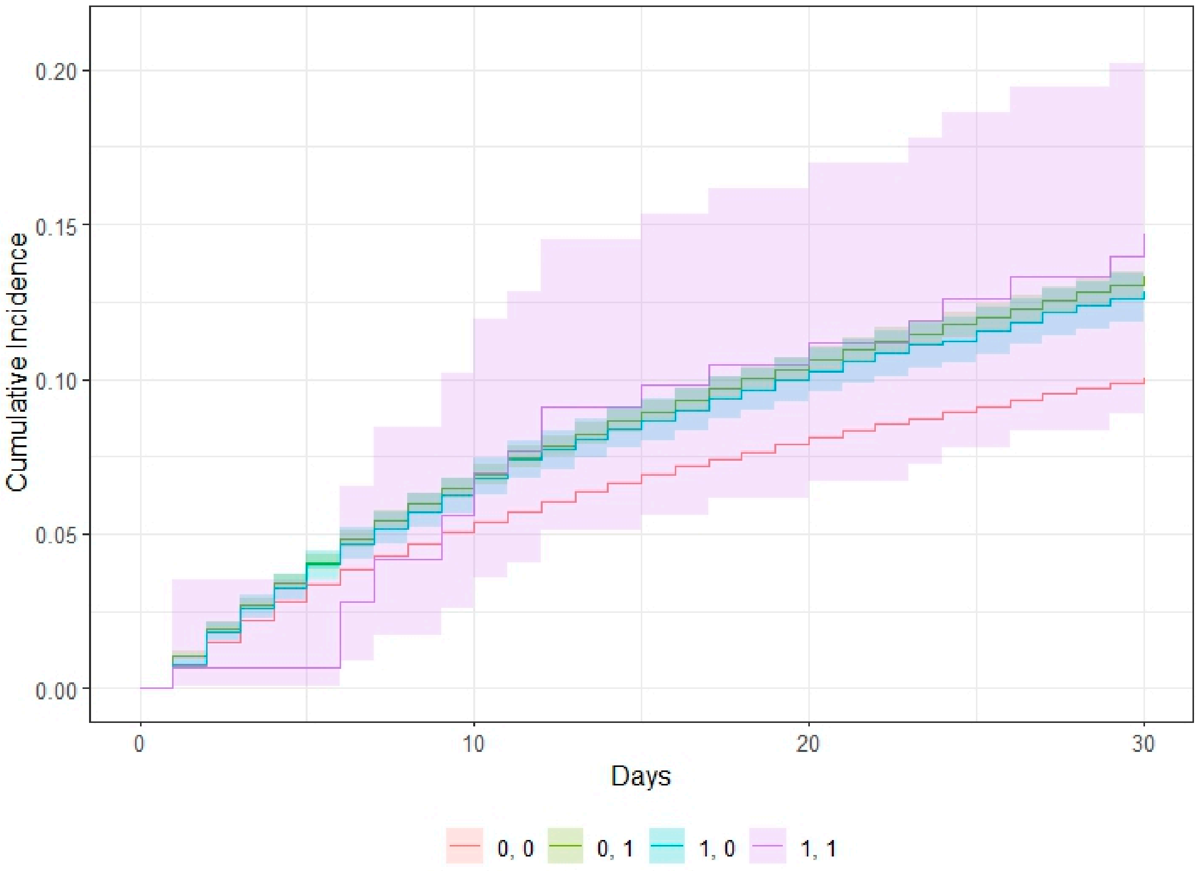

4. **Cardiac complications (cardiac tamponade, spontaneous coronary artery dissection, and pericardial effusion).**

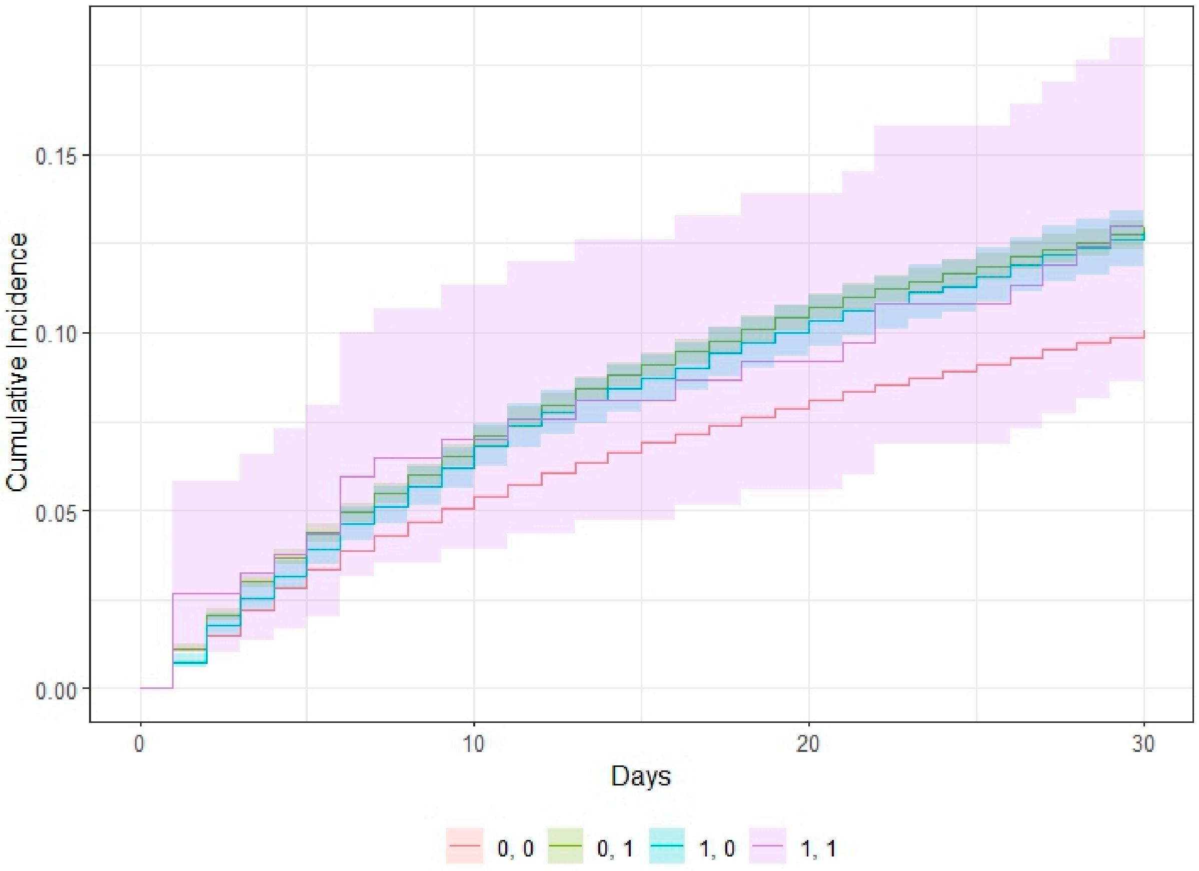

5. Acute heart failure.

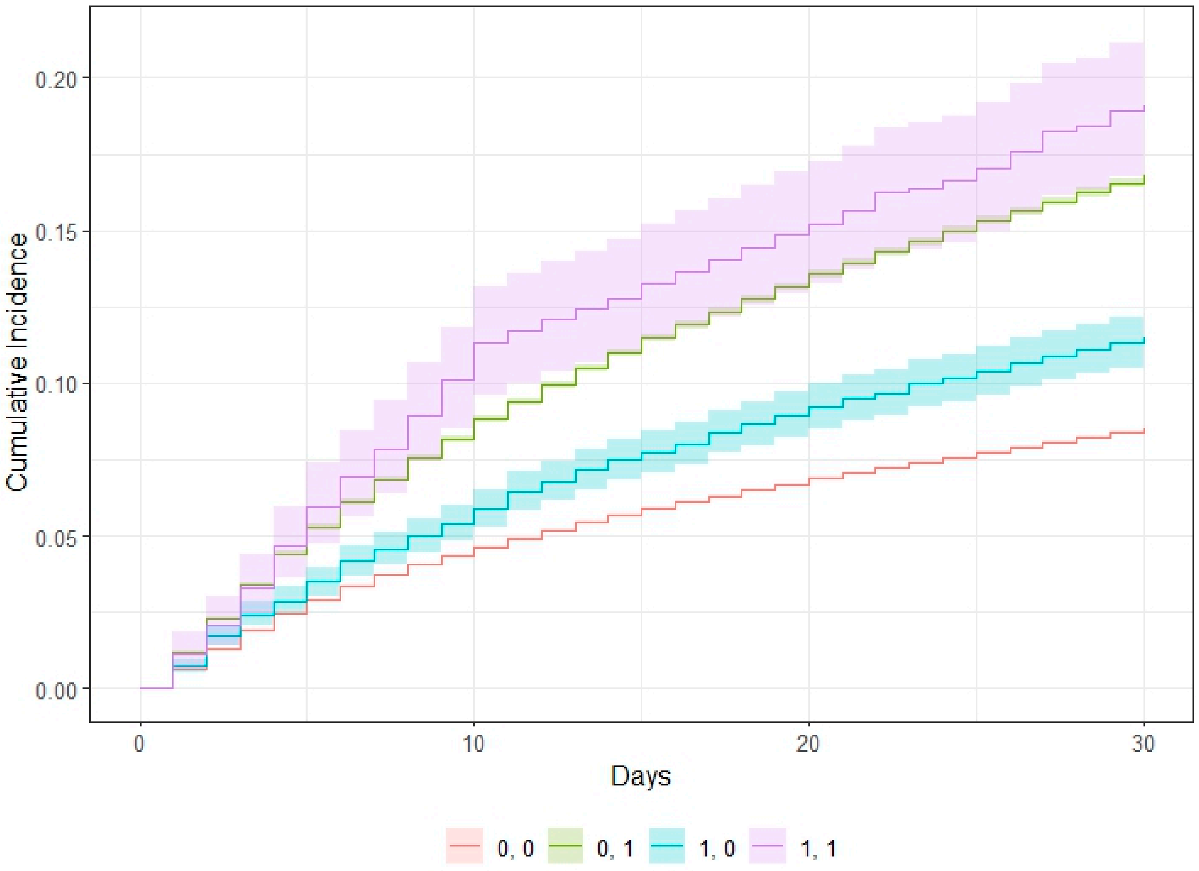

Cumulative Incidence
0.00
0.05
0.10
0.15
0.20
0
10
20
30
Days
0, 0
0, 1
1, 0
1, 1

6. **Stroke.**

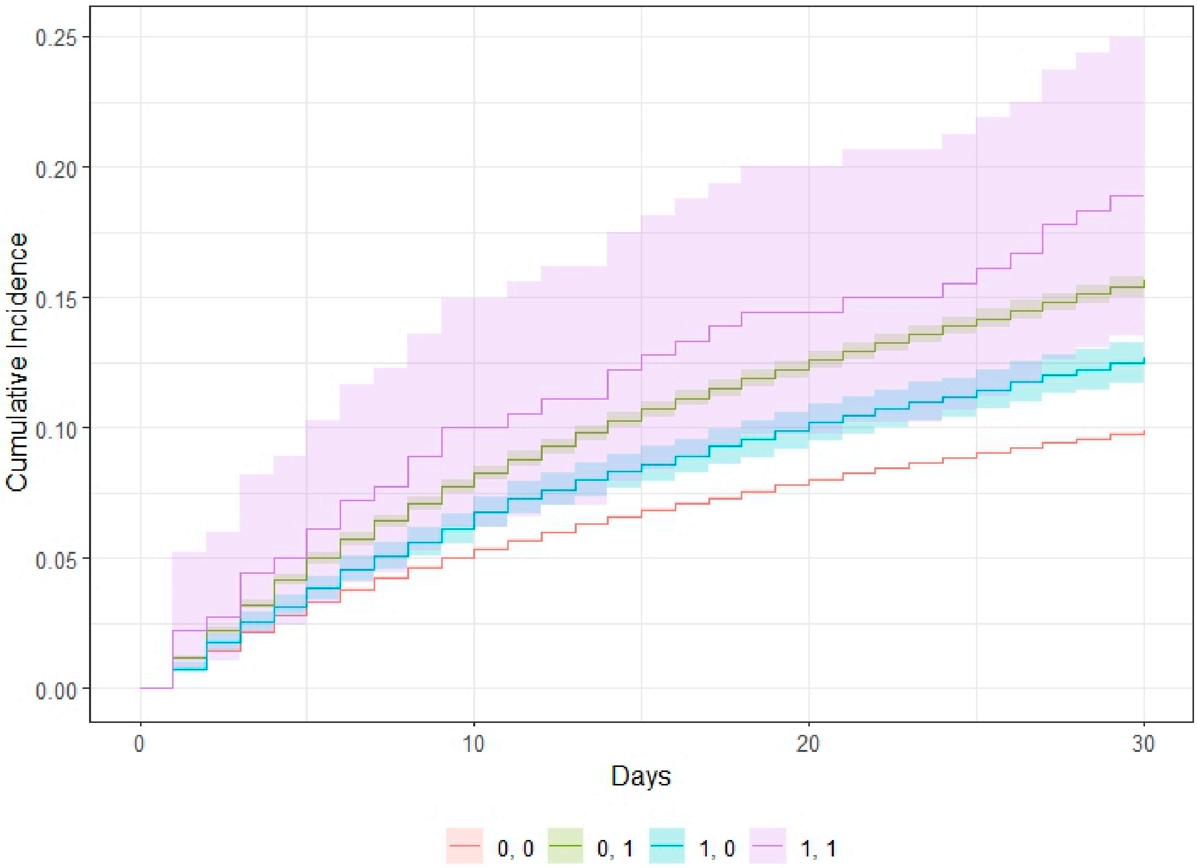

7. **Acute liver failure.**

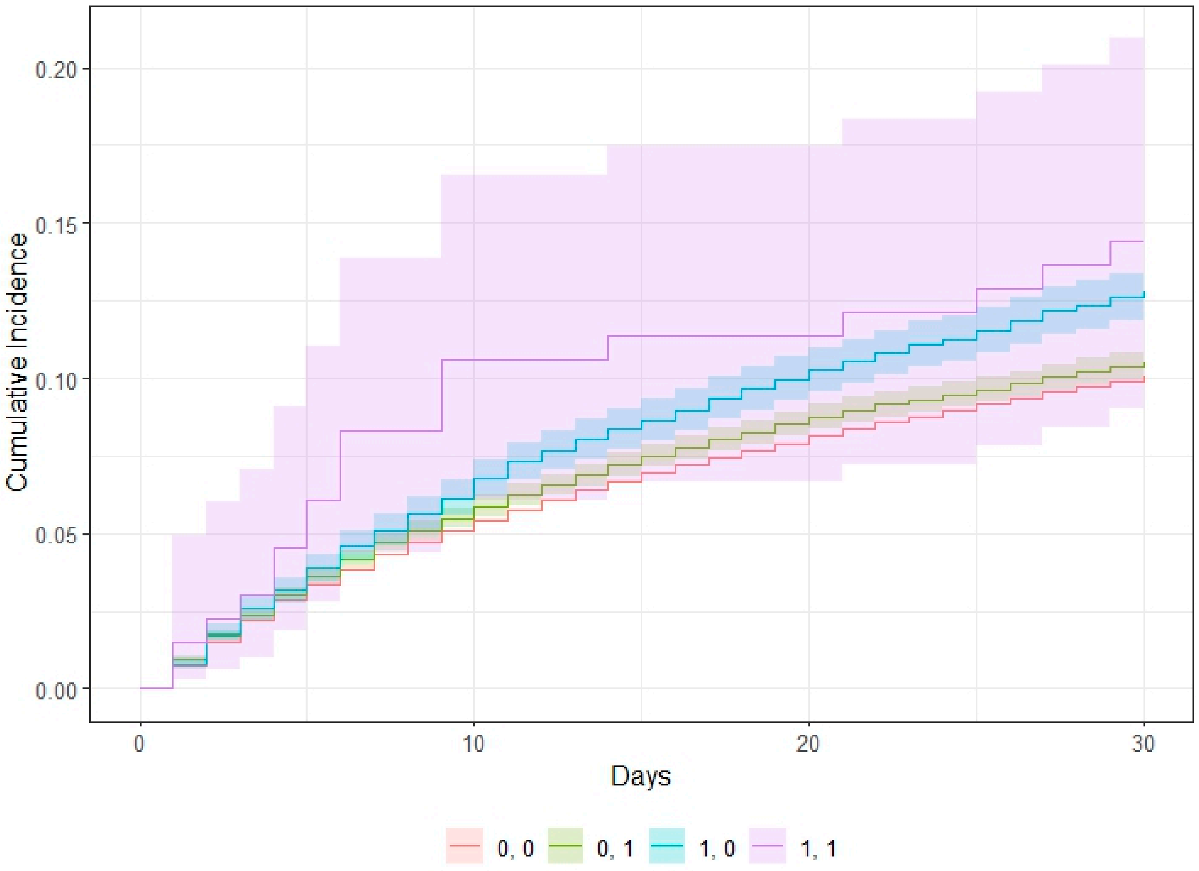

8. Mesenteric Ischemia.

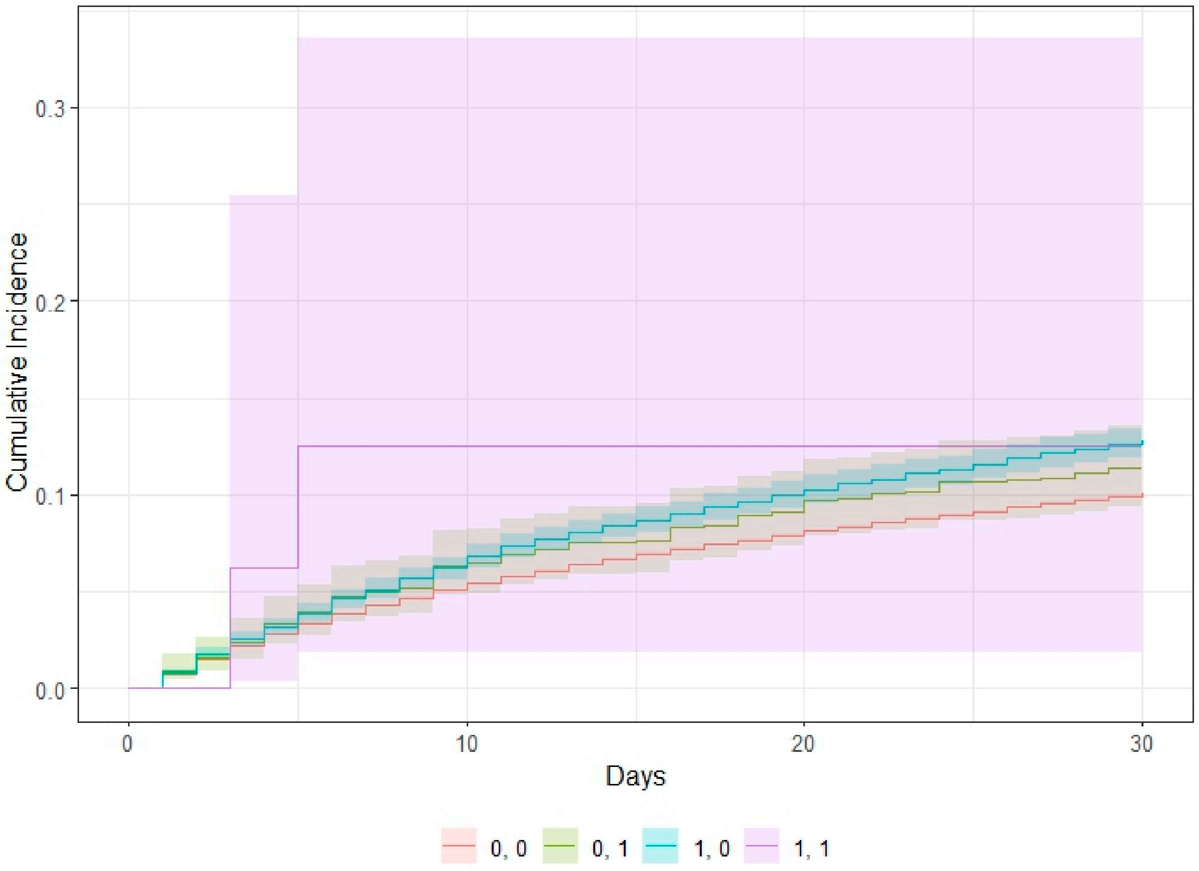

9. Packed red blood cell transfusion.

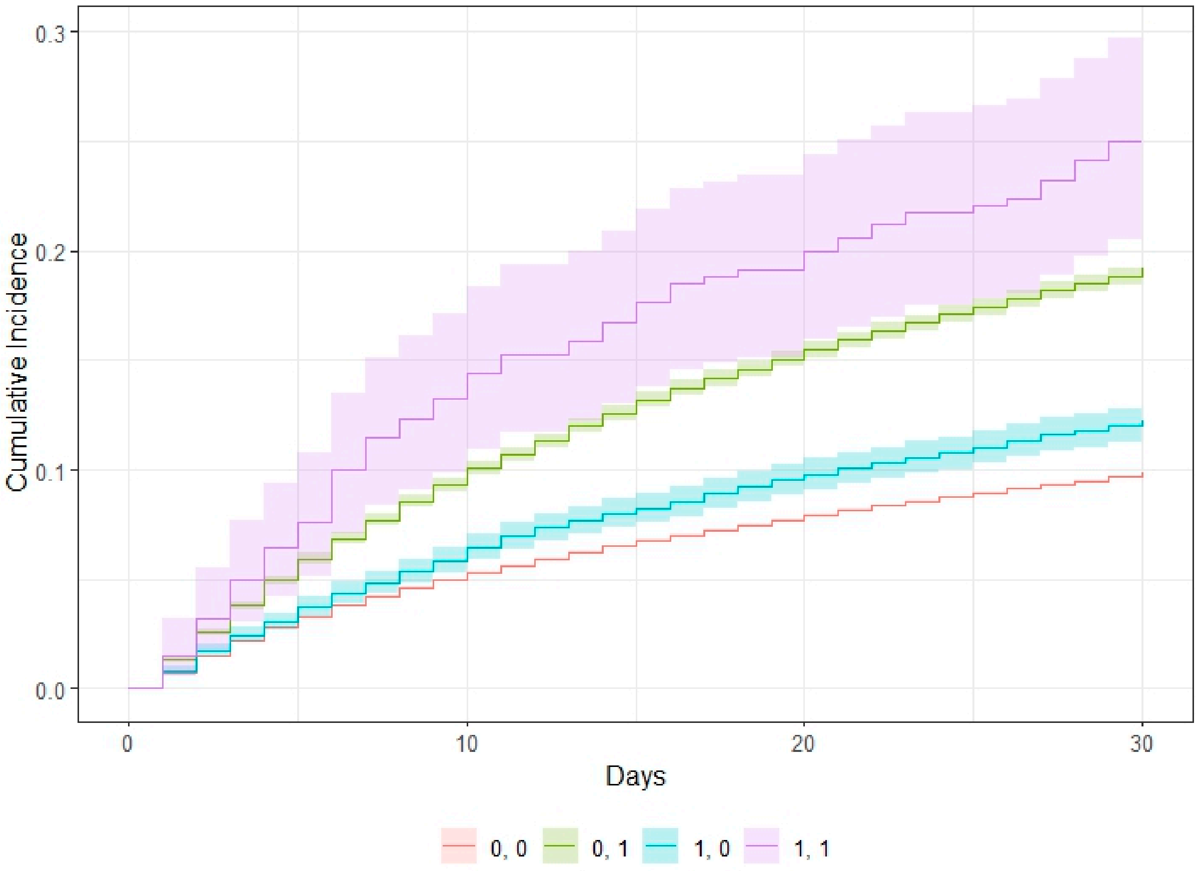

10. Other gastrointestinal complications (pancreatitis, cholecystitis, and appendicitis).

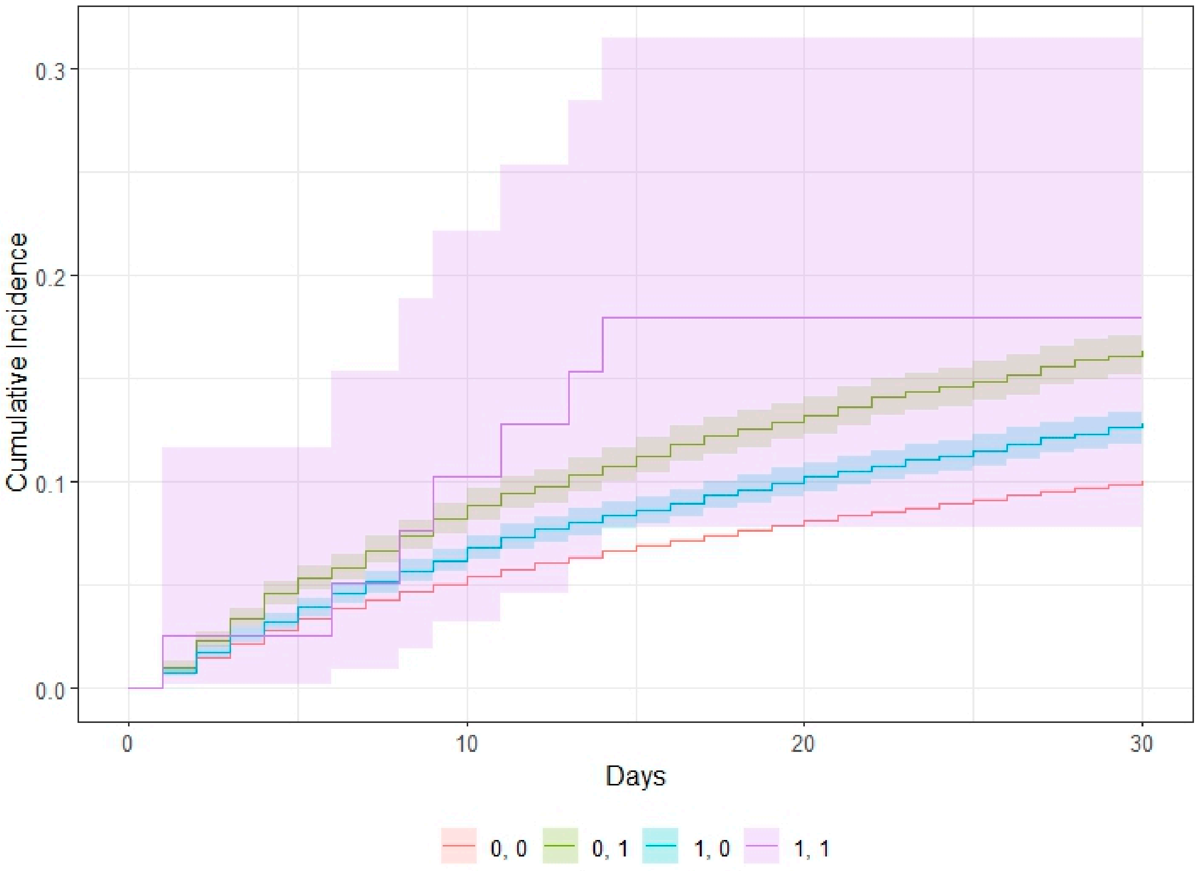

Cumulative Incidence
0.3
0.2
0.1
0.0
0
10
20
30
Days
0, 0
0, 1
1, 0
1, 1
